# Grinding the Teeth, Its Cause and Prevention*Translated from Archiv für Zahnheilkunde by R. A. MacDonald, D.D.S.

**Published:** 1917-02

**Authors:** Charles F. Bödecker


### GRINDING THE TEETH, ITS CAUSE AND PREVENTION. *

BY CHARLES F. BÖDECKER, D.D.S.

Grinding the teeth is a nervous manifestation, that has appeared in an unusual number of cases during the present trying war time, with the consequent exaggerated nervous strain and excitement. We have been forced to examine our patients with more than usual care as they are wholly unconscious of this affection. We find in initial cases that the lower incisors and upper cuspids

* Translated from Archiv für Zahnheilkunde by R. A. MacDonald, D.D.S.

are most abraded. The prominent cusps seem to afford a special incentive for the development of the habit. However, the cusps do not show the abrasion to the same extent at first as some of the teeth less protected by heavy enamel.

I have noticed this condition more or less in about twenty-five per cent. of my mature patients. When the patient's attention is called to it, he is inclined to be skeptical, and when asked to protrude the lower jaw to the position which will produce the abrasion, he immediately says it can't be, as he never bites in that position. In order to convince him it is only necessary to show him that it is not possible to produce the result shown on his teeth when the molar or grinding teeth are in normal articular relations, as at that time the incisal teeth do not occlude, and hence no abrasion is possible. Most patients deny grinding the teeth at any time, but evidently they do it unconsciously during the day or at night. There seems to be no other explanation, in view of the almost universal testimony of patients, who are so affected that they are not at all conscious of such a habit.

The cause of this abrasion of the teeth—called Bruxomania by Burchard-Inglis—may be classified in two general groups of causes.

The first class being due to unusual nervousness: (a) a permanent nervous temperament; (b) a temporary nervous irritability.

The second class may be attributed to habit: (a) such as biting the lips, finger nails, etc.; (b) concentrated thinking or working.

In the first group, nervousness due to temporary irritability is probably the greatest cause of the present epidemic of tooth abrasion. I have noticed the condition most frequently among the volunteer corps of nurses and hospital attendants. Many such people are unaccustomed to the sights of bad wounds and severe suffering and naturally they occlude the teeth with unusual force to assist in maintenance of normal equilibrium, and to enable them to go through their ordeal of "duty."

Dr. E. von Wiedekind, cites a case of a patient affected with indigestion from improper eating late in the evening, which was entirely overcome by correcting the diet. In this case the patient was greatly distressed by the grinding of the teeth, which disappeared entirely with recovery from the indigestion. The grinding is not altogether confined to the patient's unconscious periods of sleep or preoccupation, as the patients are often conscious but willing to tolerate it, as it relieves the congestion and pain tendencies.

This grinding is a useless function physiologically as it occurs when there is no food in the mouth to masticate. I have noted but a single case of bad abrasion in which the patient acknowledged that he used his teeth in a manner to produce the abrasion when eating. In this case the lower incisors were badly worn.

It is well known that patients past sixty years of age show more or less abrasion of all the teeth, but this can be accounted for by natural wear and lessened resistance because of the loss of the protecting enamel. Dentures which retain their original formation are rarely seen to be abraded. In some cases, where decay has occurred and fillings have been placed, or slight exposure of dentine has taken place, there may be seen evidences of abrasion, because of the irritation. The abrasion of patients' teeth of advanced age is not unusual, in fact, we may expect it; but it is not the characteristic abrasion produced by grinding of the teeth in younger patients, which we are discussing. It shows as much on the molars as on the incisors, as the occlusion and articular movements are at this time normal.

In the cases under discussion the molar teeth may not be affected, due probably to the fact that the mandible is protruded directly or laterally and the bite is thrown on the affected area only. Because the incisal teeth have small areas of occlusal surface, and when abrasion begins the whole force of the occluding muscles is concentrated on these small occlusal surfaces; the force is great and the wear rapid. Sometimes the teeth affected will be forced out of alignment.

In extreme cases the lower incising teeth may be worn down to the gum line, or the lingual of the upper may be cupped out, often the dentine becomes hypersensitive, and the pulps die or calcify.

The remedies which the dentist can apply are almost entirely confined to repairing the tooth wastage with fillings, inlays, etc., and in many cases these are very unsatisfactory, as they are not preventive, and may not be durable. Strong crowns or porcelain jackets have been the most successful in my experience.

Because of the difficulties in making permanent and artistic repairs it behooves us to detect the tendency at as early a date as possible, and to give our patient proper advice as to correcting the habit. It may often be necessary to inform the patient's friends of the deleterious effects of the habit, so as to enlist their co-operation in preventing the certain loss of useful teeth. If it occurs during the patient's wakeful hours it is possible to correct the habit and so prevent the result by helpful advice. But if the mischief happens whilst he sleeps it will be necessary to use some technical appliance to prevent the grinding during sleeping hours.

This can be accomplished by making a vulcanite plate which covers all the molar and bicuspid teeth while leaving the incisal teeth free. This the patient can wear at night and the teeth will be protected from the injury by preventing close occlusion. The molar teeth which come in contact with the vulcanite plate can not take injury. A few months' wearing of such an appliance will correct the habit, unless there is a local cause, which may not have been removed in the meantime.

Of course, the plate may be made of swaged gold if more comfortable for the patient, and if it is to be work in day time it would be an advantage to have it made of swaged gold, as it is less bulky and easier kept clean.

